# Multiple Sclerosis: A Disorder of Altered T-Cell Homeostasis

**DOI:** 10.1155/2011/461304

**Published:** 2011-09-15

**Authors:** David G. Haegert

**Affiliations:** Department of Pathology, McGill University, Duff Medical Building, 3775 rue University, Montreal, QC, Canada H3A 2B4

## Abstract

Uncertainty exists as to whether similar or different mechanisms contribute to the pathogenesis of different subtypes of multiple sclerosis (MS). Detailed analysis of naive T cell homeostasis shows that patients with relapsing-remitting MS (RRMS) and with primary progressive MS (PPMS) have early-onset thymic involution that causes reduced thymic output. The reduced thymic output leads to secondary peripheral homeostatic alterations in naïve CD4 T-cells, which closely mimic T-cell alterations observed in an experimental animal model of diabetes mellitus. Homeostatic T-cell receptor (TCR) signalling and proliferation of naïve T cells are induced by self-peptides. Consequently, the findings of increased TCR signalling of naïve CD4 T-cells, without increased proliferation, in PPMS, and the increased homeostatic proliferation of naïve CD4 T-cells in RRMS favour the development of autoimmunity. Thus, it seems highly likely that peripheral T-cell alterations secondary to a thymic abnormality contribute to the pathogenesis of both MS subtypes.

## 1. Introduction

MS is a chronic inflammatory and neurodegenerative disorder of the central nervous system. An ongoing issue is whether similar or different mechanisms contribute to the pathogenesis of different MS subtypes. Current opinion implicates peripheral T cells in an autoimmune response directed against CNS antigens as the pathogenetic mechanism in RRMS, but not in PPMS or in SPMS [1, 2]. Instead, some claim that CNS injury in progressive MS is due to neurodegeneration [3], or as suggested recently, to a neuroinflammatory process confined within the blood brain barrier [4]. 

The basis of autoimmunity in MS is a subject of considerable interest. Over the past twenty years, work in my laboratory investigated the possibility that a thymic abnormality contributes to the pathogenesis of MS. We initially focused on RRMS. The results of our studies of the T-cell receptor (TCR) repertoire (see below) led us to question whether RRMS patients have altered T-cell homeostasis. We also considered the possibility that PPMS patients share T-cell homeostatic alterations with RRMS patients. Several studies suggest that T-cell homeostatic alterations contribute to autoimmunity. The thymus has been shown to have an important role in the regulation of experimental autoimmune encephalomyelitis (EAE) [5]. Peripheral homeostatic T-cell responses to lymphopenia have a central role in the development of experimental autoimmune diabetes [6] and in patients after islet cell transplants [7]. In rheumatoid arthritis, one group of investigators reported homeostatic peripheral T-cell alterations, possibly secondary to reduced thymic output although direct evidence of a thymic alteration was not obtained [8]. As summarized below, we have direct evidence of reduced thymic output not only in RRMS but also in PPMS. This reduced thymic output leads to peripheral homeostatic responses and T-cell activation in both MS subtypes. It is proposed here, analogous to lymphopenia-induced autoimmunity [6], that reduced thymic output results in a homeostatic response that contributes to the pathogenesis of both RRMS and PPMS. That is, both subtypes of MS are disorders of T-cell homeostasis.

## 2. Studies of the TCR Repertoire in RRMS

In 1993, Utz et al. reported that antigen-stimulated T-cells from RRMS patients have major shifts in their overall TCR diversity, that is, TCR repertoires [9]. This report led us to question whether TCR repertoire shifts occur in unstimulated T-cells from RRMS patients and whether these shifts could contribute to the pathogenesis of RRMS. In order to address this possibility, we analyzed freshly isolated naive CD4 T-cells, which by definition have not been antigen-stimulated. We reasoned that changes in these T cells would be independent of the immune response in RRMS and precede the onset of RRMS. We studied identical twins discordant for RRMS. In comparisons with healthy identical twin pairs, both the affected twins and their healthy cotwins had TCR repertoire shifts, specifically in the complementarity-determining 3 (CDR3) regions of several TCR V beta gene segments [10]. Since we analyzed naïve T-cells, the findings suggested that these shifts precede RRMS and likely predispose to the development of RRMS. Importantly, it seemed unlikely that these shifts were sufficient to initiate MS by themselves, as both the healthy and the affected members of the discordant twin pairs had TCR repertoire shifts [10]. 

We considered several possible explanations for the TCR repertoire shifts. Firstly, we excluded genetic differences between twin pairs, including HLA differences, as explanations for the TCR repertoire shifts (see [10] for a detailed discussion). In this regard, a genetic influence on CDR3 repertoires is highly unlikely; 90% of TCR diversity is randomly generated by nucleotide additions within the CDR3 region during thymopoiesis and these additions are independent of genetic background [11]. Secondly, a stochastic basis is improbable, since both members of the discordant MS twin pairs shared the TCR repertoire shifts. Thirdly, and a more likely explanation is that unknown environmental factors affected both members of each discordant twin pair so as to alter TCR repertoire formation in the thymus; we have no direct evidence to support this explanation. Since the thymus has a central role in naïve T-cell generation and T-cell homeostasis (reviewed in [12]), we hypothesized that MS is a disorder of T-cell homeostasis.

## 3. T-Cell Homeostasis: A Brief Summary

Homeostasis is the capacity of a biological system to maintain its equilibrium by physiological mechanisms in response to change [13]. T-cell homeostasis is tightly regulated and includes a balance between thymic output, which decreases exponentially with increasing age, delivery of death and survival signals, naïve T-cell differentiation into memory cells, and peripheral T-cell proliferation. This T-cell proliferation is an important contributor to T-cell homeostasis in healthy individuals, as it helps maintain T-cell numbers in the face of thymic involution [14].

## 4. Reduced Thymic Output in RRMS and in PPMS

**Figure 1:**
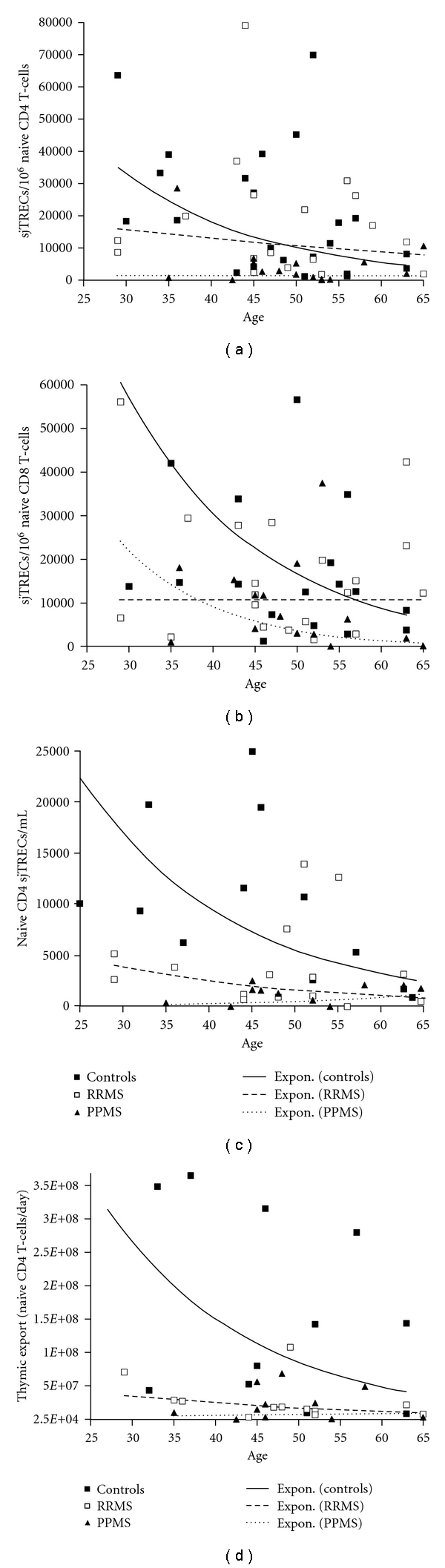
sjTRECs in controls and patients. (a) Naïve CD4 sjTREC frequencies (sjTRECs/10^6^ naïve CD4 T-cells) decreased significantly with age only in controls (*P* = 0.037). (b) Naïve CD8 sjTREC frequencies decreased with age only in controls (*P* = 0.037). (c) Total naïve CD4 sjTREC numbers decreased exponentially with age only in controls (*P* = 0.048). Median sjTRECs were higher in controls than in RRMS (*P* = 0.03) or in PPMS (*P* = 0.007) with a trend towards higher sjTREC numbers in RRMS versus PPMS (*P* = 0.055). (d) Thymic export (naïve CD4 T-cells/day) decreased with age only in controls (*r* = −0.564, *P* = 0.05). Both RRMS (*P* = 0.005) and PPMS patients (*P* = 0.004) had significantly reduced median daily thymic export compared to controls.

 Signal joint T-cell receptor excision circles (sjTRECs) are often quantified as a measure of thymic output [14–26]. sjTRECs form as by-products of TCR*α* gene rearrangement during late thymopoiesis. These sjTRECs are cytoplasmic circular DNA fragments (episomes) and cannot replicate during mitosis [14, 15]. Consequently, reduced sjTREC frequencies, that is, reduced sjTRECs in constant cell numbers (e.g., sjTRECs/10^6^ cells), could be due either to reduced thymic output or to increased peripheral T-cell proliferation [14, 15]. In RRMS, various investigators report reduced sjTREC frequencies in peripheral blood mononuclear cells (PBMC) or in T subsets. As one might expect from the difficulty in interpreting sjTREC frequencies, some claim reduced thymic output whereas others raise the possibility of increased peripheral T cell proliferation in RRMS [18–22]. An important reason for the different interpretations is that none of these studies included a direct measure of thymic output, which excludes the contribution of peripheral T cell proliferation. Naïve T cells contain the vast majority of sjTRECs whereas memory T cells contain very few sjTRECs [14]. Memory T cell numbers increase with age [27] and might also vary with the magnitude of the chronic immune response in MS. Consequently, any demonstration of patient-control differences in the sjTREC content of PBMC or total T cells could reflect, in part, differences in memory T-cell numbers between patients and controls. In order to avoid these difficulties in interpreting sjTRECs, we isolated naïve T subsets and studied their sjTREC content. In order to exclude possible treatment influences on sjTRECs, we only studied RRMS patients in remission; none had been treated with corticosteroids, immunomodulatory agents in the year prior to the study, and none of the patients had ever received cytotoxic agents. Since most RRMS patients receive some form of treatment, we could not identify sufficient patients to control for disease duration. No patient had overt evidence of thymic disease such as myasthenia gravis. In one study, we found that RRMS patients under, but not over, age 40 have significantly reduced sjTREC frequencies compared to age-matched controls [23]. In a second study of both RRMS and PPMS patients, we observed that the sjTREC frequencies of naive CD4 and naïve CD8 T cells decrease exponentially with age in healthy controls [24], as shown in Figures 1(a), and 1(b). This finding is consistent with several reports indicating that thymic output decreases with increasing age [14, 15]. In contrast to controls, naive T-cell sjTREC frequencies did not show a significant decrease with age in RRMS or in PPMS. Instead, the younger RRMS and PPMS patients had lower sjTREC frequencies than controls of a similar age, and these frequencies remained at low levels with increasing age of the patients [24]. These findings suggested that both RRMS and PPMS patients have reduced thymic output.

Since sjTRECs are influenced not only by thymic output but also by T cell division [14, 15], we sought direct evidence of reduced thymic output in MS patients. Total sjTREC numbers in the naïve T cell pool, for example, sjTRECs/mL of peripheral blood, are unaffected by peripheral T-cell proliferation and consequently provide a superior measure of thymic output [25]. Total naive CD4 T-cell sjTRECs were significantly reduced in RRMS and PPMS patients compared to controls (see Figure 1(c)), indicating reduced thymic output in both MS subtypes [24]. To extend this work, we exploited a mathematical model, which provides the first reported quantitative measure of thymic export (T-cells/day). This model takes into account naïve CD4 T-cell sjTREC content and the absolute number of naïve CD4 T-cells and identifies and excludes the contribution of T-cell proliferation, measured by expression of the proliferation marker Ki-67, to naïve CD4 T-cell sjTREC content [26]. Application of this model demonstrated that both RRMS and PPMS patients have reduced thymic export of naïve CD4 T cells/day compared to controls, and that thymic export is low in young patients from both patient groups and remains low with increasing age (see Figure 1(d)). Surprisingly, thymic export of naïve CD4 T-cells/day was lower in PPMS than RRMS [24]. Taken together, the quantitative data indicate that both RRMS and PPMS patients have a major thymic alteration with early-onset thymic involution and resulting reduced thymic output [23, 24].

## 5. Increased Naïve CD4 T-Cell Survival Signals in MS

The long-term maintenance of function and survival of peripheral naïve Tcells depends upon TCR signalling via self-peptide-MHC class II molecules and upon IL-7 receptor (CD127) signalling via IL-7, which in turn induces expression of the anti-apoptotic molecule Bcl-2 [12]. We questioned whether survival signals are increased in RRMS and PPMS. In one study, we found apparently increased expression of Bcl-2 by CD31-negative (CD31neg) cells from RRMS patients versus controls [23]; CD31-positive naïve CD4 T cells include CD4 recent thymic emigrants whereas CD31neg cells are distant in origin from the thymus [16, 28]. We found increased expression of Bcl-2 by both naïve CD4 and CD8 T cells from PPMS patients compared to controls [24]. These findings suggest increased delivery of survival signal in both patient groups, which may partly compensate for the reduced thymic output in these patients, that is, helps maintain the size of the peripheral naive T-cell pools. Increased Bcl-2 expression may also have relevance for MS susceptibility; overexpression of *β*-arrestin 1, a positive regulator of Bcl-2 expression, increases susceptibility to EAE, and CD4 T cells in MS have increased expression of *β*-arrestin 1 [29].

## 6. Increased Naive T-Cell Proliferation and TCR Signalling in MS

The magnitude of proliferation of early (CD3-CD4-CD8-) thymocytes is the main determinant of thymic output. Proliferation of early thymocytes decreases progressively with age, which explains the progressive age-associated decreasing thymic output that occurs in healthy individuals [30, 31]. We used two strategies to assess peripheral homeostatic proliferation; in all studies, we analyzed naïve T-cell expression of the proliferation marker Ki-67, which is expressed in all cells in the G1, S, G2, and M phases of the cell cycle [32]; in one study, we quantified naïve CD4 T-cell *β*TRECs, which are a byproduct of early thymocyte proliferation [30]. In an early study, we reported increased Ki-67 proliferation levels in naïve CD8 T cells from RRMS patients versus controls [18]. In a later study, we reported progressive age-associated reduction of naïve CD4 T-cell *β*TRECs in RRMS patients versus controls. This finding suggested that RRMS patients have increased naïve CD4 T-cell homeostatic proliferation [23]; an increase in thymocyte proliferation seems an unlikely explanation of the reduced *β*TRECs, as such proliferation is inconsistent with thymic involutional processes [30, 31]. We also attempted to use a more direct measure of thymocyte proliferation, the sj/*β*TREC ratio; to obtain this ratio some quantify either 6 or 10 *β*TRECs, extrapolate to 13 *β*TRECs, and then calculate the sj/*β*TREC ratio [31, 33]. After quantifying six *β*TRECs in cohorts of RRMS patients and age-matched controls, we found that the individual *β*TREC levels differed significantly from one another. We concluded in our laboratory that calculations of sj/*β*TREC ratios would not provide accurate, direct measures of thymocyte proliferation [23]. Detailed comparisons of Ki-67 expression levels in naïve CD4 T-subsets from RRMS patients and controls did show, however, increased naïve CD4 T-cell proliferation in RRMS, predominantly in the CD31-positive cells having the highest levels of CD31 expression (CD31hi cells or CD4 recent thymic emigrants—for details, see [23]) but also in CD31neg cells. Our proliferation data suggest that increased peripheral T-cell proliferation may partly contribute to reduced sjTRECs in RRMS, as suggested in other reports [18, 22], but, as noted in Section 4, reduced sjTRECs in RRMS are mainly due to reduced thymic output. 

**Figure 2:**
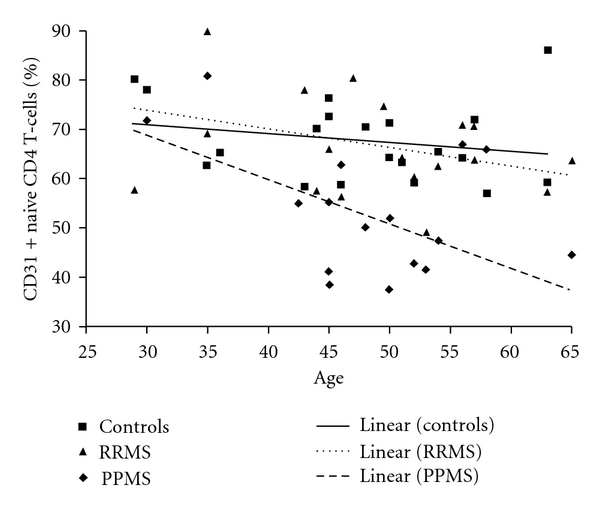
Naive CD4 T-cell expression of CD31. The proportion of naïve CD4 T-cells expressing CD31 showed a significant decrease with age only in PPMS (*P* = 0.031).

Several reports emphasize that TCR signalling of CD31-positive naive CD4 T cells induces loss of CD31 expression, which is accompanied by increased naive CD4 T-cell proliferation [28, 34]. We analyzed the proportion of naive CD4 T-cells expressing CD31 as a potential indicator of increased proliferation in PPMS, in comparisons with RRMS and controls. An age-associated decrease in this proportion only in PPMS (see Figure 2) [24] initially suggested increased naive CD4 T-cell proliferation in PPMS compared to the other two groups. However, Ki-67 expression did not differ in PPMS from controls or RRMS patients [24]. Also, if increased proliferation had occurred in PPMS, naïve CD4 T-cell sjTREC frequencies should also have decreased with age, but that did not occur [24]. A recent study has showed that TCR signalling does not induce total loss of CD31 from the T-cell surface but rather cleavage and shedding of a portion of CD31 containing the epitope recognized by the commonly used anti-CD31 antibody [35]. Accordingly, our data indicate increased TCR signalling of naïve CD4 T cells in PPMS without increased proliferation.

## 7. Conclusions

The direct evidence for early-onset thymic involution and reduced thymic output in RRMS, described above, is supported by the indirect evidence of reduced thymic output from other laboratories [19–22]. To my knowledge, ours is the only group to report reduced thymic output in PPMS. One limitation to our studies is that we have no definite evidence as to the basis of the reduced thymic output in MS although our TCR repertoire studies raise the possibility that some unknown environmental factor/factors target(s) the thymus in RRMS (see above). Direct analysis of thymic export suggests that thymic alterations occur early in life in RRMS and PPMS, either at the time of MS onset or before and that the thymic “defect” persists throughout life, as indicated by low but constant levels of thymic export with increasing age. In other words, our findings suggest an early onset of thymic involution in MS. One study of immunoablation/autologous stem cell transplantation as a treatment of MS showed that the post transplant MS patients “rebooted” their immune system such that the thymus produced new T cells having a diverse TCR repertoire [36]. This finding is not inconsistent with our results; RRMS patients over age 40 continue to generate recent thymic emigrants, which contain the majority of naïve T cell TCR diversity [37] although this generation is at significantly lower levels than age-matched controls [23].

A critical question is whether reduced thymic output has relevance to the pathogenesis of MS. Both RRMS and PPMS patients have increased naive CD4 T-cell expression of Bcl-2 [23, 24]. Increased expression of this survival signal may partly compensate for reduced thymic output in RRMS and PPMS by helping to maintain the size of the naïve T-cell pool. As noted in Section 5, increased expression of this anti-apoptotic molecule may also increase susceptibility to MS. Homeostatic proliferation induced by self-peptide MHC constricts the TCR repertoire and inevitably expands autoreactive T cells, particularly autoreactive CD31neg naive CD4 T cells [28, 34]. The increased homeostatic proliferation in RRMS is analogous to the homeostatic proliferation that initiates autoimmune diabetes in a mouse model [6], that is, it seems logical to implicate this process in the pathogenesis of RRMS. TCR signalling causes partial cleavage and shedding of CD31 from naive CD4 T cells [35]. This shedding abrogates the activity of CD31-associated immunotyrosine-based inhibitory motifs (ITIMs) [35]. In PPMS, increased TCR signalling of naive CD4 T cells, presumably by self-peptides, could activate self-reactive naive CD4 T cells and initiate autoimmune responses. Since naive CD4 T cells have a central role in initiating immune responses, including autoimmune responses in RRMS [38], the peripheral TCR signalling/proliferative responses to reduced thymic output likely initiate autoreactivity in PPMS and RRMS. If this view is correct, it remains unresolved as to why PPMS patients show minimal responses to anti-inflammatory and immunosuppressive agents [39] and why PPMS patients have a paucity of new MRI lesions [40]. Obviously, peripheral homeostatic naive T-cell responses to early-onset thymic involution will persist throughout life in PPMS and RRMS in order to maintain the naive T-cell pool. Natural regulatory T cells (nTregs) are included within the naive CD4 T subset [41] and show disturbed development and function in SPMS [42]. One possibility in PPMS is that early-onset thymic involution alters nTregs, either quantitatively or qualitatively, so that CNS injury is continuous without relapses, as suggested by one study of T-cell regulation in PPMS [43]. Further analysis of regulatory T-cell function in PPMS is essential to address this issue.
